# Investigating the effect of an identified mutation within a critical site of PAS domain of WalK protein in a vancomycin-intermediate resistant *Staphylococcus aureus* by computational approaches

**DOI:** 10.1186/s12866-021-02298-9

**Published:** 2021-09-02

**Authors:** Neda Baseri, Shahin Najar-Peerayeh, Bita Bakhshi

**Affiliations:** Department of Bacteriology, Faculty of Medical Sciences, Tarbiat Modares University, Tehran, Iran

**Keywords:** Vancomycin, Antimicrobial Drug Resistance, Per-Arnt-Sim domain kinase, Sensor Histidine Kinase, Molecular Structures, Computational Biology

## Abstract

**Background:**

Vancomycin-intermediate resistant *Staphylococcus aureus* (VISA) is becoming a common cause of nosocomial infections worldwide. VISA isolates are developed by unclear molecular mechanisms via mutations in several genes, including *walKR*. Although studies have verified some of these mutations, there are a few studies that pay attention to the importance of molecular modelling of mutations.

**Method:**

For genomic and transcriptomic comparisons in a laboratory-derived VISA strain and its parental strain, Sanger sequencing and reverse transcriptase quantitative PCR (RT-qPCR) methods were used, respectively. After structural protein mapping of the detected mutation, mutation effects were analyzed using molecular computational approaches and crystal structures of related proteins.

**Results:**

A mutation WalK-H364R was occurred in a functional zinc ion coordinating residue within the PAS domain in the VISA strain. WalK-H364R was predicted to destabilize protein and decrease WalK interactions with proteins and nucleic acids. The RT-qPCR method showed downregulation of *walKR*, *WalKR*-regulated autolysins, and *agr* locus.

**Conclusion:**

Overall, WalK-H364R mutation within a critical metal-coordinating site was presumably related to the VISA development. We assume that the WalK-H364R mutation resulted in deleterious effects on protein, which was verified by *walKR* gene expression changes.. Therefore, molecular modelling provides detailed insight into the molecular mechanism of VISA development, in particular, where allelic replacement experiments are not readily available.

**Supplementary Information:**

The online version contains supplementary material available at 10.1186/s12866-021-02298-9.

## Introduction

Vancomycin-intermediate resistant *Staphylococcus aureus* (VISA), which causes a wide range of life-threatening infections, has been recovered worldwide [1, 2]. Nevertheless, the molecular mechanism of VISA development is incompletely understood. The molecular studies on clinical or laboratory-derived VISA isolates have confirmed that VISA phenotype is caused by the diverse nonsynonymous single nucleotide polymorphisms (nsSNPs). However, only a few of these mutations have been verified by allele swapping or complementation experiments. The most relevant nsSNPs reported in VISA isolates are mutations in *walKR*, *graSR,* and *vraTSR* two-component systems (TCSs) [3].

The WalKR system, specific TCS to low G + C Gram-positive bacteria, is the only essential TCS in *S. aureus* viability [4, 5]. The WalKR plays a role in cell wall biosynthesis, virulence, and antibiotic resistance due to its extensive interaction network, such as interaction with *graSR*, *vraSR*, *rpo*, *agr*, and autolysins genes (*atlA*, *sle1*, and *lytM*) [4, 6, 7]. The *walKR* mutations may cause destructive regulation of these genes that can arise identical features among VISA isolates, such as reduced autolytic activity and virulence [8].

In *S. aureus*, the PAS domain (amino acids: from 261 to 375) is conserved among members of *Staphylococcus* species and contains four critical and highly conserved residues (His 271, Asp 274, His 364, Glu 368) as metal-binding sites, which directly bind to a Zinc ion (Zn^2+^) [9].

In *Bacillus subtilis* (*B. subtilis*), the role of the cytoplasmic PAS (PAS^CYTO^) domain in the localization of WalK in the division septum is confirmed [10]. Recently, structural and functional analyses of Zn^2+^-binding residue 271 within the PAS domain of *S. aureus* WalK showed that the Zn^2+^-binding site regulates *S. aureus* WalKR [9]. On the other hand, a substitution mutation in H271 was recently reported in a VISA strain [11]. In *B. subtilis*, residue D274 was confirmed to be related to the regulatory pathways of peptidoglycan synthesis [12].

Missense mutations can disrupt drug-bacterial interaction and protein function via affecting protein stability and interaction with biological molecules such as protein and nucleic acid. The previously deposited crystal structures of the desired bacterial species in the mutation site are utilized to analyze the effect of nsSNP on the protein structure and interactions of protein using molecular computational approaches. These methods for predictions of these impacts usually are used to understand the mechanism of human genetic disease and drug resistance to infections such as *Mycobacteria* infections [13–16]. Despite the role of WalKR in VISA development, the molecular modelling of the mutations site and computational approaches in VISA strains requires improved understanding.

Therefore, besides the gene expression and complementation experiments to understand the VISA molecular mechanisms, the importance of knowledge about protein structure mapping of mutations and predicting the impact of the mutation on protein function using molecular computational approaches can provide new insight into the mechanisms underlying VISA development and help to design novel therapeutic strategies [17].

In the present study, we detected mutation WalK-H364R, which locates in the Zn^2+^-binding residue of the PAS ^CYTO^ domain, in a laboratory-derived VISA strain compared to its wild-type strain. This mutation was also previously reported in a *S. aureus* strain after nisin selection [18]. In the present study, we attempted to generate a VISA mutant via the selection by vancomycin in a VSSA (vancomycin susceptible *S. aureus*) strain. The reverse transcriptase quantitative PCR (RT-qPCR) method was used to compare the expression of TCSs (*walkR*, *vraTSR*, *graSR*) genes in the VISA strain compared to the wild-type strain. Using the Sanger sequencing method, we compared the complete sequence of *walK* and *walR* genes between VSSA and VISA strains, which showed *walKR* expression changes. After detection of WalK-H364R mutation within the PAS domain of the VISA strain, this study aimed to predict the possible effects of this mutation on protein function, protein stability, and protein interactions using computational approaches. We supported *in silico* results by comparing phenotypic features and gene expression of *agr* RNA III and *walK*-regulated autolysis genes in the VISA and VSSA strains. Details of the mutation effects can evaluate whether the identified mutations have the potential to change on protein and possible variations of the bacteria characteristics. These findings help to understand the mechanism of VISA development that is an urgent need to identify new drug targets or to design new drugs.

## Results

### *In vitro* development, stability, and isogenicity of VAN-I mutant

After a serial adaptation process, VAN-I strain (vancomycin MIC = 8 μg/ml) was selected as a VISA strain from a clinical VSSA strain (VAN-S; vancomycin MIC = 1 μg/ml). In this process, the vancomycin MIC of VAN-S was increased to 2, 4, and 8 μg/ml after 6, 10, and 18 days, respectively.

The vancomycin MIC in VAN-I mutant was constant on the vancomycin-free medium over five passages that revealed stability of VISA phenotype at the tested time point.

**Fig. 1:**
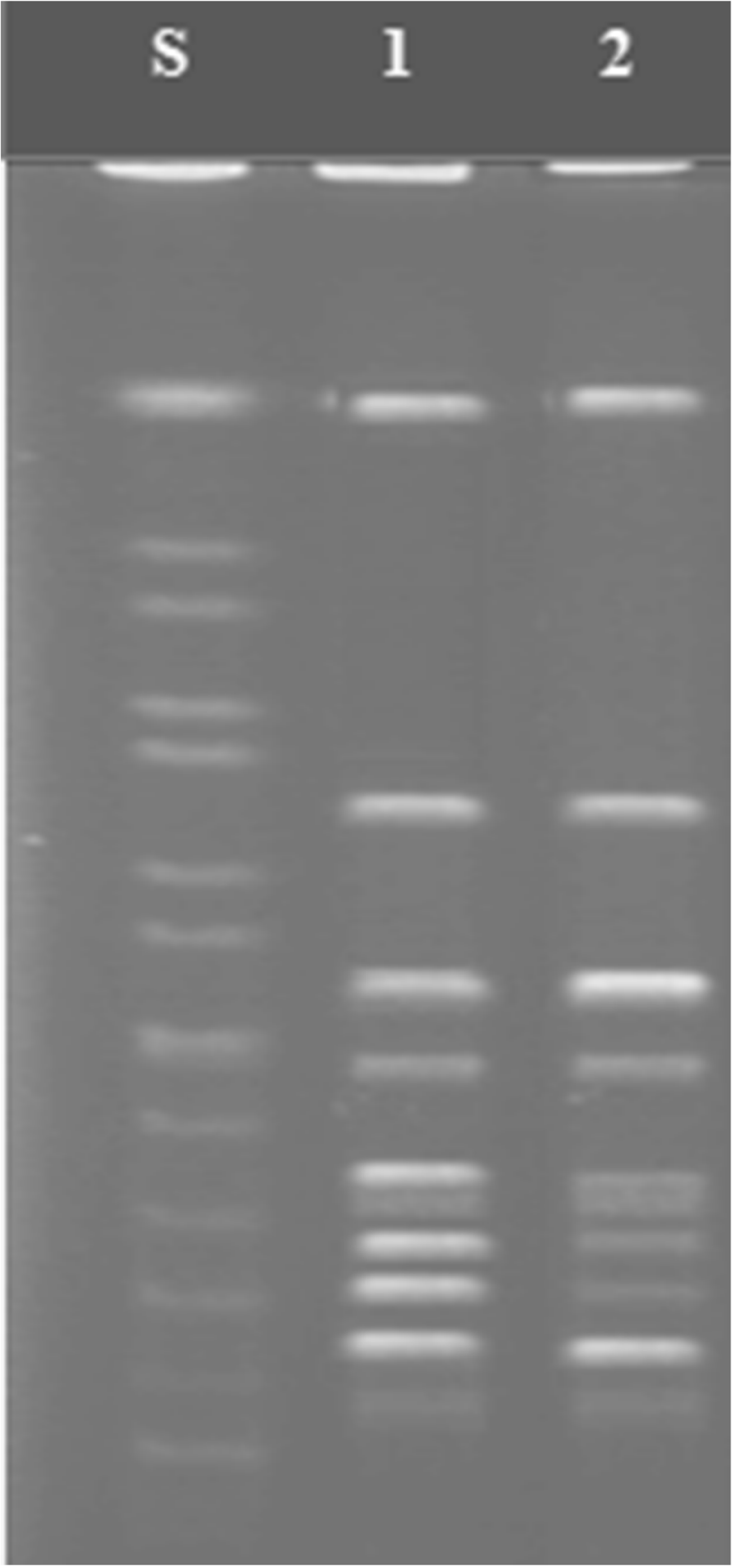
PFGE profile of parent (VAN-S) and mutant (VAN-I) strains. Labels from left to right indicated: S = Standard (*Salmonella* ser. Braenderup H9812), 1 = VAN-S, 2 = VAN-I. The banding pattern of VAN-S and VAN-I is the same (the number and location of bands). The isogenicity of the VISA mutant and its wail-type strain was confirmed by the PFGE result

The same PFGE profile confirmed the isogenicity of VAN-S and VAN-I (Fig. 1).

### Transcriptional changes in VAN-I mutant

**Fig. 2:**
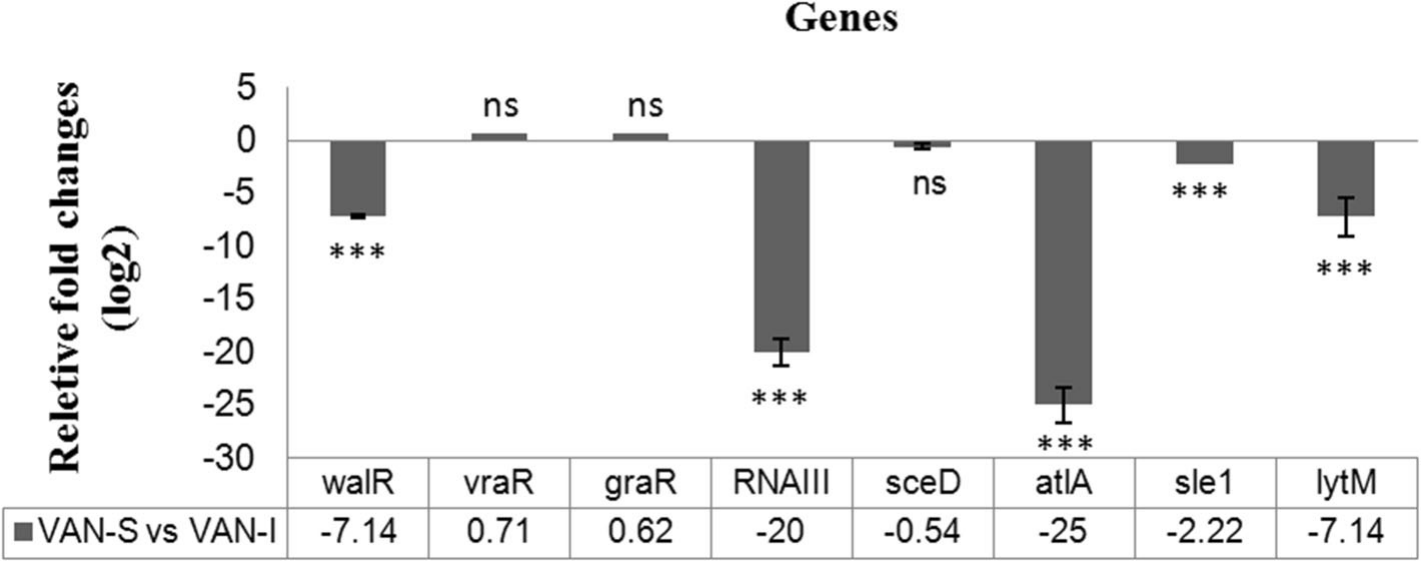
RT-qPCR analysis to quantify the expression of *walR*, *vraR*, *graR*, *agr* RNA III, *sceD*, *atlA*, *sle1*, and *lytM* genes in VAN-I mutant compared to VAN-S parental strain. The gyrA was used as the standard internal gene. The results are presented in the relative fold-change. Error bars show standard deviation (±SD) for at least three independent experiments. The symbol *** indicates a significant statistical difference (*P <* 0. 001) on gene expression in VAN-I versus VAN-S using REST2009 software, whereas ns means no significant difference

RT-qPCR analysis to quantify the expression changes of *walR*, *vraR*, *graR*, *agr* RNAIII*, sceD*, *atlA*, *sle1*, and *lytM* genes are presented in Fig. 2. The analysis of transcriptional profiles of *walKR*, *vraTSR*, and *graSR* systems indicated that the expression of *walKR* (0.14-fold; 7.14 times) was significantly (*P ≤* 0.05) downregulated in VAN-I mutant. There was no significant statistical difference in the gene expression of *vraTSR* and *graSR* systems between VAN-I and VAN-S strains (Fig. 2). Furthermore, the comprising of *walKR*-dependent peptidoglycan hydrolase genes (*lytM*, *atlA*, *sle1*, and *sceD*) showed the downregulation (*P ≤* 0.05) of *lytM* (0.14-fold; 7.14 times), *atlA* (0.04-fold; 25 times), and *sle1* (0.45-fold; 2.22 times) genes in VAN-I mutant. No significant change was observed in the expression of *sceD* gene. A notable reduction (*P ≤* 0.05) in *agr* RNAIII expression (0.05-fold; 20 times) of VAN-I strain showed reduced activity of *agr* locus.

### Identification of the nsSNP mutation within Zn^2+^-binding site of Walk in VAN-I mutant

**Fig. 3:**
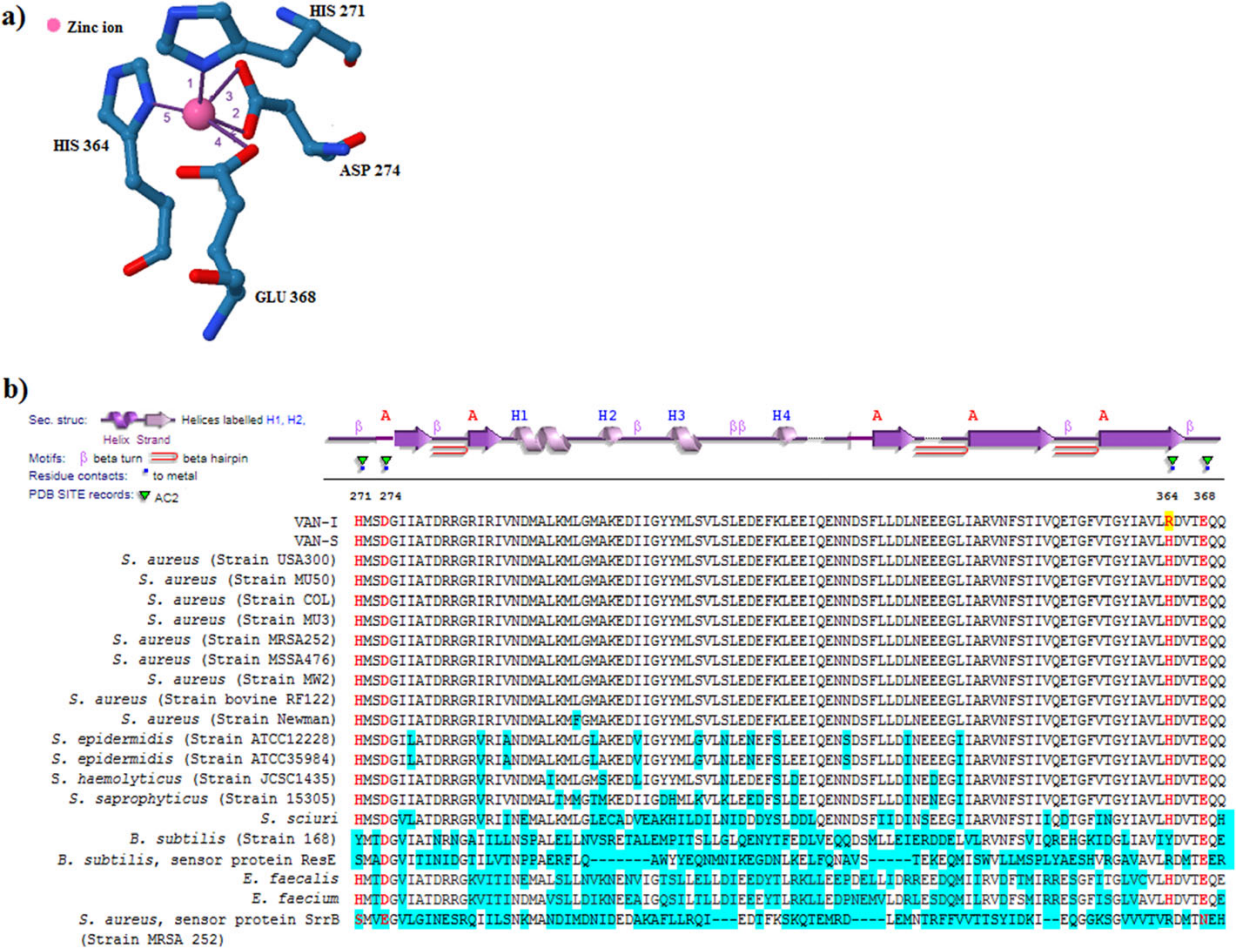
The location of Zn^2 +^ −binding sites in WalK protein. **(A)** The Zn^2+^-binding residues (HIS 271, ASP 274, HIS 364, and GLU 368) of WalK protein coordinated with Zn^2+^. Blue rods show the Zn^2+^-binding residues. The coordinating bonds are shown with purple lines and numbers (1, 2, 3, 4, and 5). The image is generated by Protein-Ligand Interaction Profiler (PLIP) web service (https://projects.biotec.tu-dresden.de/plip-web/plip/index). PDB ID 4mn6 was used as a wild-type template. **(B)** Alignment of residues 271-370 of WalK protein in *S. aureus* with other staphylococci, bacilli*,* and enterococci*.* Zn^2+^-binding residues are shown in red colour. The mutated residue in VAN-I is highlighted in yellow. The nonconserved residues are highlighted in blue

After aligning the *walK* in wail-type (VAN-S) and mutant (VAN-I) strains, one nsSNP was detected in VAN-I mutant (*walK* nucleotide 1091, base change: A to G). In this mutation, Histidine 364 was replaced by Arginine (WalK- H364R) in WalK protein. Mapping of mutation within WalK protein revealed that WalK-H364R was located in the Zn^2+^-binding site within the PAS^CYTO^ domain [9]. The Zn^2+^-binding sites of WalK protein are displayed in Fig. 3a.

### Conservation of Zn^2+^-binding sites in WalK protein

After alignment of WalK sequences (in the selected region) among staphylococci, enterococci, and bacilli genera, the potential conservation of Zn^2+^-binding residues (including H364) among WalK protein of staphylococcus species and enterococci were confirmed (Fig. 3b).

### Comparison of protein structural mapping of previously reported nsSNPs in WalK protein

**Fig. 4:**
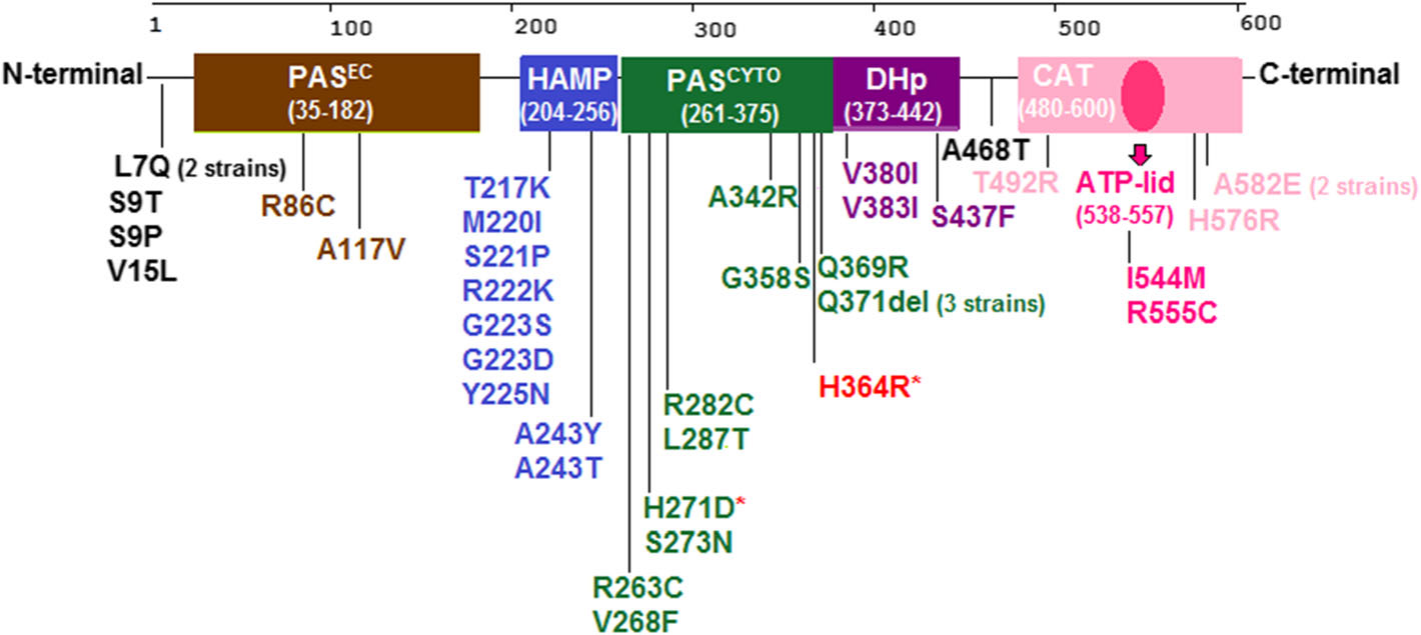
Schematic map of WalK protein domains and location of nonsynonymous single nucleotide polymorphism (nsSNP) in sensor protein kinase WalK of *S. aureus*. H364R detected in the present study is indicated in red colour. Other included nsSNPs were previously reported in vancomycin nonsusceptible *S. aureus* strains. The nsSNP that occurred in the same domain are shown with the same colour. The nsSNP with the red symbol (*) indicates the presence of a mutation in a Zn^2+^-binding site. PAS^EC^: extracellular PAS domain; HAMP: present in Histidine kinases, Adenyl cyclases, Methyl-accepting proteins, and Phosphatases domain; PAS^CYTO^: cytoplasmic PAS domain, DHp: dimerization and histidine phosphorylation domain; CAT: catalytic and HATPase (ATP-binding) domain

The protein structural mapping of 38 nsSNPs reported in previous studies in WalK of vancomycin non-susceptible *S. aureus* strains is shown in Fig. 4. The results displayed that most nsSNPs were located in the PAS^CYTO^ domain (12 nsSNPs) and HAMP domain (9 nsSNPs).

Among the studies reviewed, we observed that one nsSNP (H271D) within the Zn^2+^-binding site (residue 271) of WalK in a VISA strain [11].

### Protein stability change upon mutation H364R in WalK

We analyzed the impact of mutation H364R within a zinc^2+^-binding residue of WalK protein using the DUET method. DUET method predicted that the nsSNPs could directly influence protein stability via changes in ΔΔG (kcal/mol) value.

Overall, the presence of mutation H364R was estimated to have a destabilizing effect on WalK protein stability. The results are displayed in Table 1 and include the combined DUET prediction and the mCSM and SDM individually predicted changes.

**Table 1:** Predicted stability changes upon mutation H364R using DUET prediction web server. The negative ΔΔGs (kcal/mol) indicate the destabilizing effect of mutation H364R

Method Wild-type	mCSM Predicted Stability Change (ΔΔG: kcal mol)	SDM Predicted Stability Change (ΔΔG: kcal/mol)	DUET Predicted Stability Change (ΔΔG: kcal/mol)
**4mn5-chain A**	−1.4	−1.0	−1.371
**4mn5-chain B**	−1.478	−1.19	−1.484
**4mn6-chain A**	−1.364	−1.58	−1.423
**4mn6-chain B**	−1.478	−1.19	−1.484

### Protein-protein complex affinity change upon mutation H364R in WalK

The mSCM-PPI2 predicted that the protein-protein affinity of WalK destabilized (Reduced affinity) upon mutation H364R. The ΔΔG ^affinity^ against 4mn5 was −0.045 kcal/mol in chain A and − 0.084 in chain B. The ΔΔG ^affinity^ against 4mn6 was −0.069 kcal/mol in chain A and − 0.106 kcal/mol in chain B. The inter-residue non-covalent interaction network of VAN-I mutant and wild-type (PDB ID 4mn6) are displayed in Supplementary Fig. S1.

### Protein-nucleic acid binding affinity change upon mutation H364R in WalK

The mCSM-NAv2 displayed that protein-nucleic acid binding affinity was reduced. Therefore**,** WalK-H364R had a destabilizing effect on protein-nucleic acid interaction. ΔΔG^affinity^ was 0 kcal/mol against both PDB structures in chain A and chain B. ΔΔG^stability effect^ against 4mn5 and 4mn6 was −1.4 kcal/mol and − 1.364 in chain A and − 1.478 kcal/mol and − 1.53 in chain B, respectively.

### Prediction of the impact of mutation H364R in WalK on protein function

SIFT estimated that substitution at position 364 from H to R is predicted to be tolerated with a score of 1.00 and median sequence conservation: 3.02.

Due to the importance of the mutation site, the result of SIFT prediction was surprising.

The SIFT alignment results in FASTA format showed that WalK sequences are homologous to *S. aureus* SsrB and ResE in *B. subtilis* in some residues (Fig. 3b). The alignment of WalK showed that an R364 residue (similar to the amino acid substituted in H364R) in protein sensor kinases SrrB in *S.aureus* and ResE in *B. subtilis* was matched with residue 364 (Arginine) of VAN-I mutant (Fig. 3b). Therefore, this similarity could lead to predicting a tolerated effect of WalK-H364R by the SIFT method. We searched the binding sites in SsrB protein using PLIP (Protein-Ligand Interaction Profiler) web service (https://projects.biotec.tu-dresden.de/plip-web/plip/index) (input PDB ID: 6PAJ; data not shown). No Zn^2+^-binding site was detected in SrrB. Furthermore, residue 348 in SsrB showed no evidence of a ligand-binding site. Therefore, this residue has a different function in SrrB compared to it in WalK. This result showed a low accuracy of the SIFT method in classifying nsSNPs within binding sites of *S. aureus* WalK.

### Phenotypic changes: decreased doubling time, autolytic kinetic, and hemolytic activity in VAN-I

In this study, VAN-I mutant had longer doubling times (DT) than in VAN-S (DT^VAN-S^ = 27.28 min versus DT^VAN-I^ = 45.47 min).

**Fig. 5:**
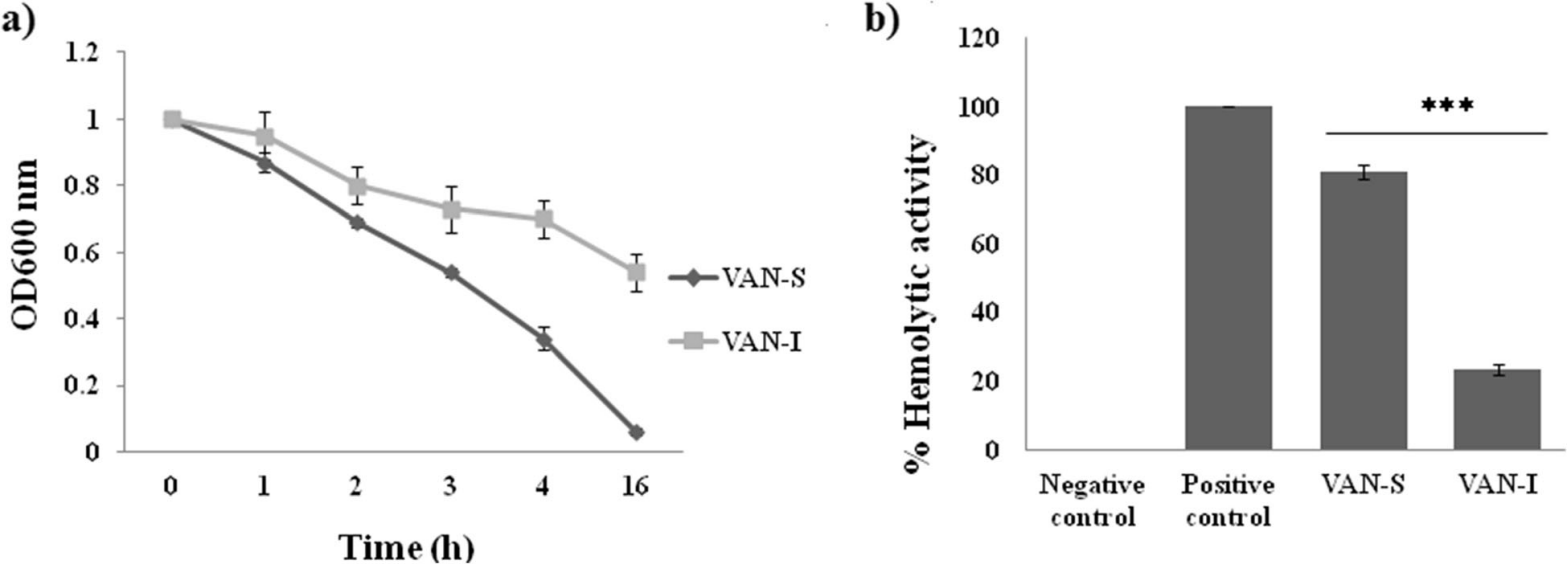
Comparative phenotypic characteristics of an isogenic VAN-S/VAN-I pair (with missense mutation H364R). The error bars indicate the standard deviation (±SD) of values obtained by spectrophotometer in three independent tests. A *P* value ≤0.05 was considered as a significant difference between VAN-S and VAN-I. **(A)** The kinetic data set of autolysis of the *S. aureus* culture (in 20 ml BHIB) that washed (two times) and adjusted to an OD_600_ of 1 (in 10 ml cold distilled water supplemented with 0.1% Triton X-100) was measured at OD_600_ in the tested time points. There was a statistically significant difference (*P* < 0. 01) in the autolysis kinetic activity of VAN-I versus VAN-S (paired-sample Student’s *t*-test) at each tested point. **(B)** The percent of hemolysis of 300 μl of human red blood cells (RBCs) diluted in 10 ml PBS in exposure to *S. aureus* culture (adjusted to an OD_600_ of 0.3 in BHIB) was measured at OD_543_ using the following formula: (A_543_ of the sample ˗ A_543_ of negative control) / (A_543_ of positive control ˗ A_543_ of negative control) × 100. Negative control: diluted RBC in PBS without bacteria; Positive control: Triton X-100; the symbol (***) indicates a significant difference (*P* < 0.001) using the two-tailed Wilcoxon signed-rank test

Autolytic activity at the tested time points in VAN-I was significantly decreased (*P ≤* 0.05) compared to that in VAN-S (Fig. 5a).

Furthermore, the hemolytic activity of VAN-I was decreased compared to that in VAN-S (23.43% versus 81.03%) (Fig. 5b).

## Discussion

Although most reported nsSNPs in VISA strains occur in regulatory TCSs (e.g. *walKR*), the molecular mechanisms of resistance to vancomycin in VISA remain unclear [3]. In this study, we have reported a VISA strain with a nsSNP, which occurred in a functional residue within the PAS^CYTO^ domain of WalK protein. This residue of WalK protein binds to a specific metal ligand.

The complementation experiments have shown that sometimes only one nsSNP in the *walKR* system can lead to reduced vancomycin susceptibility. For instance, Walk-G223D or WalK-Q371del could convert VSSA to hetero-resistant VISA (hVISA) [6, 19]. WalK-Q371del was located in the PAS^CYTO^ domain.

Recently, the X-ray crystal structure of the PAS^CYTO^ domain of *S. aureus* WalK has revealed four Zn^2+^-binding sites, including His 271, Asp 274, His 364, Glu 368 within the PAS^CYTO^ domain. Zn^2+^ is tetrahedrally-coordinated by four Zn^2+^-binding sites [9]. In the present study, WalK-H364R occurred in residue His 364 in VISA strain VAN-I. This nsSNP was also previously reported to involve nisin resistance in an *S. aureus* strain after nisin selection [18]. Nisin disrupts the cell membrane as a primary target. Furthermore, it inhibits the peptidoglycan biosynthesis in Gram-positive bacteria through specific lipid II interaction [20]. Lipid II is also a target for vancomycin [21]. Coates-Brown et al. suggested that WalK-H364R could limit pore formation and nisin interaction with lipid II through the increased cell wall thickness, which is a common feature in VISA strain [18]. This hypothesis also provides new insight into the importance of WalK-H364R in developing vancomycin resistance in VISA. High conservation of Zn^2+^-binding sites among staphylococci WalK and the importance of residue H364 in *walKR* regulation following binding to Zn^2+^ ligand can support this hypothesis (Fig. 3b).

In addition to the regulation of cell wall metabolism, the WalKR system regulates pathogenesis and host inflammatory response. Decreased or attenuated virulence in VISA strains is a stealth strategy to evade the host immune and persistent infection [8]. Using the *Galleria mellonella* model, virulence analysis of the clinical VISA strains and *walK* mutants (e.g. WalK-G223D) have shown that WalKR mutation can influence pathogenicity and susceptibility to vancomycin in *S. aureus* [6]. Additionally, Allelic exchange experiments to verify the identified *walK* mutations in VISA strains using comparative genomics of VSSA/VISA clinical (before and after *in vivo* vancomycin treatment failure in patients) or laboratory-derived pairs showed attenuated virulence. Those changes include reduced autolytic and hemolytic activities and increased biofilm formation [3, 8, 22]. Here, *walKR* and *walKR*-related autolysins, including *sle1*, *lytM*, and *atlA*, were downregulated in VAN-I compared to those in VAN-S. Moreover, we showed phenotypically reduced autolysis and hemolysis that might be related to dysfunctional *walKR.* The *walK* mutations also conferred downregulation of *agr* locus in VISA strains [6, 8]. Agr locus is a global regulatory system in *S. aureus* that is involved in virulence mechanisms [23]. In the present study, we also observed decreased *agr* RNAIII expression as an effector molecule of the *agr* locus. Collectively, these events could be related to H364R, which might negatively regulate the *walKR* regulon. Subsequently, it led to increased resistance to vancomycin. Thus, the nsSNPs in Zn^2+^-binding sites likely induce different effects on the regulation of *walKR* regulon. We used computational approaches to support these hypotheses.

Molecular mapping and computational approaches have been used for predicting the effects of nsSNP on the development of drug resistance, including isoniazid and rifampicin resistance in *Mycobacterium tuberculosis* [14], rifampin resistance in *Mycobacterium leprae* [15], and carbapenem resistance in *Acinetobacter baumannii* [16]. However, studies on VISA development are limited to allele swapping and complementation experiments [3]. Although these studies are needed to confirm the effect of nsSNPs in VISA development, the knowledge on molecular modelling of nsSNPs helps to a better insight into the molecular mechanism of VISA development. These approaches are easy and fast, and the only necessity is the published crystal structure of the desired bacterial species in the mutation site.

Here, the molecular computational analyses showed that substitution H364R in the Zn^2+^-binding site of the PAS ^CYTO^ domain decreased the stability, flexibility, and interactions (with proteins and nucleic acids) WalK. The destabilizing effect of mutations on protein leads to significant disruption of protein function or regulation [24]. Here, decreasing the affinity of WalK interactions (with proteins and nucleic acids) in VAN-I mutant was confirmed by the downregulation of walKR and *walKR*-regulated autolysins. Thus, H364 can be a destructing mutation in the WalK and involve in VISA development. However, the allelic replacement experiments and future studies need to complete confirmation. These results showed the importance of nsSNP in the Zn^2+^-binding site of the PAS ^CYTO^ domain of WalK.

The molecular models have suggested that the activation of WalK histidine kinase in *S.aureus* induces through the specific interaction of the PAS^EC^ domain with D-Ala-D-Ala residue, which leads to phosphorylation of the WalR [5]. Recently, Monk et al. have suggested that Zn^2+^-binding sites within the PAS^CYTO^ domain have a regulatory role within *S. aureus* WalK. They showed that substitution in Zn^2+^-binding residue 271 (WalK-H271Y) led to inhibition of Zn^2+^ binding and activation of *walKR* regulon. H271Y mutant showed the increased hemolysis, *atl* expression, and vancomycin sensitivity [9]. However, the genome sequencing of a VSSA/VISA pair revealed a mutation in the Zn^2+^-binding site (residue 271: WalK-H271D), which was associated with increased resistance to vancomycin in the VISA strain [11]. Moreover, substitution in Zn2 + −binding (H271Y mutant) displayed reduced size and pigmentation of colonies, reduced growth rate, and increased doubling time similar to VISA strains [9]. In the present study, an overview of the protein structure mapping of several previously reported nsSNPs in VISA strains indicated that WalK nsSNPs frequently occurred in the PAS^CYTO^ domain [6, 11, 19, 25–27]. However, we observed that nsSNP could detect in all WalK protein domains. These results explain the diversity of nsSNPs in VISA strains. This diversity may occur due to the differences in the genetic backgrounds of isolates [28], which needs future analyses.

Finally, the main limitations of the present study need to be mentioned; first, the predicted results of *in silico* analysis should be verified by strong wet laboratory experimental evidence. Second, whole-genome sequencing and allelic replacement experiments were not performed. Therefore, some phenotypic and transcriptomic changes observed in the VISA strain might not be due to the mutation in the *walK* gene, but might be due to nsSNPs in other parts of the genome. Third, we have observed that sometimes there may be exceptions to the accuracy of test results. For instance, the low accuracy of the SIFT method in classifying mutation in the metal-binding site of protein was observed.

## Conclusion

In the present study, WalK gene sequencing of a VSSA/VISA pair reveals a mutation H364R in a critical coordinating Zn^2+^ site of the PAS^CYTO^ domain. Molecular computational approaches show that this mutation can induce destabilizing effects on WalK protein function in VISA strain through decreasing stability and interactions of protein-protein and protein-nucleic acid. The *walKR*, *agr* RNA III, and *walKR*-related genes that downregulated in VISA strain have verified these results. Mutation H364R might play a role in VISA development due to the importance of its location in protein structure. Collectively, molecular mapping and computational approaches can be beneficial in understanding the mechanism of VISA development and designing novel antimicrobial drugs.

## Material and Methods

### Bacteria parental strain and growth conditions

A clinical MRSA (Methicillin-resistant *S. aureus*) strain was isolated from a burned patient in Motahhari Hospital, Tehran, Iran, in December 2018. This strain was called VAN-S and served as the parental vancomycin susceptible strain (with a MIC value of vancomycin equal to 1 μg/ml). VAN-S strain harboured type III SCC*mec* and *agr* type III.

*S. aureus* was cultured within the brain heart infusion (BHI) agar (BHIA) and BHI broth (BHIB) (Ibresco, Italy) at 35 ± 2 °C with shaking at 200 rpm otherwise stated.

### *In vitro* selection of VISA strain

One VISA mutant was selected by vancomycin from one *S. aureus* clinical strain and then compared with its VSSA wild-type to study the mechanism of VISA development [3].

Here, vancomycin 500 mg vial (Dana Co, Iran) was used for *in vitro* selection of VISA strain (VAN-I). It was reconstituted in sterile distilled water to achieve the desired concentrations, including 1/4, 1/2, 1, and 2× vancomycin MIC of VAN-S strain (0.25, 0.5, 1, and 2 μg/ml, respectively).

Overnight culture of VAN-S strain was adjusted to 5 × 10^5^ CFU/ml concentration into 3 ml of BHIB containing 1/4 × MIC of vancomycin. After 24 h, the culture was serially passaged into BHIB containing 1/2, 1, and 2 × MIC of vancomycin. Then, the process was repeated from 1/4 × MIC of vancomycin. The cultures in each step were speared on BHIA plates to maintain vancomycin tolerance colonies.

This process was repeated for 50 days. The MIC of vancomycin was recorded every 24 h.

### Stability of VISA phenotype and isogenicity between parental and mutant strains

After VAN-I mutant selection, it was cultured on vancomycin-free BHIA plates over five passages to evaluate the stability of VAN-I.

To confirm the isogenicity of VAN-S and VAN-I, pulsed-field gel electrophoresis (PFGE) was performed using SmaI endonuclease (Takara, Japan), as previously described [29]. 1% agarose gel containing digested DNA samples were run on the CHEF-Mapper (Bio-Rad Laboratories, CA) for 19 h at 6 V/cm and a field angle of 120°, with switch times of 5 and 40 s at a temperature of 14 °C.

### Determination of minimum inhibitory concentration of vancomycin

The broth macro dilution method was used to determine the MIC of vancomycin according to the 2018 CLSI guidelines [30]. The cation-adjusted Mueller-Hinton broth medium (Merk, Germany) was used for the MIC assay.

### RNA extraction and reverse transcriptase quantitative PCR (RT-qPCR)

The RNA was extracted from VAN-S and VAN-I cells in the mid-logarithmic phase using the GeneAll Hybrid-R™ RNA isolation kit (Geneall Biotechnology, Korea) with DNase I treatment (Thermo Fisher Scientific, USA) of 1 μg of RNA template (final concentration: 0.1 μg/μl).

After cDNA synthesis (Yekta Tajhiz Azma, Iran), RT-qPCR was run on a Rotor-Gene Q (Qiagen, Hilden, Germany) using RealQ Plus 2x Master Mix SYBR Green (Ampliqon, Denmark), and primers (Pishgam Biotech, Iran) (see Supplementary Table S1) to amplify *agr* RNAIII and TCSs genes, including *walR*, *vraR*, *graR*. If there was a gene expression change in these TCSs (see result section), the RT-qPCR method was also performed to amplify TCSs-regulated genes. Here, the expression change of *atlA*, *lytM*, *sle1*, and *sceD* genes was examined following changes in *walKR* expression. The *gyrA* was used as the internal control. The samples were run at least in triplicate assays.

### DNA extraction, gene sequencing, and protein mapping of the mutation

Genomic DNA from overnight cultures of VAN-S and VAN-I was extracted using the Gene Transfer kit (Pioneers, Iran).

PCR was performed to amplify TCS genes that showed gene expression changes by the RT-qPCR method (see result section). Therefore, *walK* and *walR* genes were amplified using the primers (see Supplemental Material Table S1 online) within a T100TM Thermal cycler (Bio-Rad, USA).

The PCR products were confirmed by electrophoresis on a 1% agarose gel, purified, and then sequenced (by Microsynth AG Co, Switzerland) using the Sanger sequencing method (forward and reverse reads).

The results were checked using Chromas software (V2.6.6; https:// technelysium.com.au/chromas.htm), then aligned using allele ID software (V6.00; Premier Biosoft, USA) and also the BLAST database.

After detection of nonsynonymous substitution mutation in WalK protein, we determined the protein domain mapping [9] of mutation detected in this study as well as in some previously studies that reported WalK mutations in vancomycin nonsusceptible *S. aureus* strains, including T492R [31]; R555C [32]; A243Y, S9T, V15L, H271D, A342R, G358S [11]; L7Q [11, 33]; A582E [11, 27]; Q371del [11, 19, 25]; A117V, A468T, R222K [34], Y225N [33], S221P [35], R86C, I287T [27], I544M [36], Q369R, M220I [26]; V380I, V383I, S9P, A243T, R282C, T217, G223S, H576R, S437F [28]; R263C, S273N [25]; G223D, V268F [6].

Furthermore, the conservation of the mutation site among staphylococcal species and other closed genera was examined using the alignment of WalK sequences. The following bacterial strains were used for sequences alignment: *S. aureus* (USA300, MU50, COL, MU3, MRSA252, MSSA476, MW2, bovine RF122, and Newman), *Staphylococcus epidermidis* (ATCC 12228, and ATCC 35984), *Staphylococcus haemolyticus* (JCSC1435), *Staphylococcus saprophyticus* (15305), *Staphylococcus sciuri* (K3), *B. subtilis* (168), *Enterococcus faecalis* (V583), *Enterococcus faecium* (AUS0085).

### Protein stability analysis

The sequence alignment of WalK protein VAN-S was performed to obtain the crystal structure of *S. aureus* WalK protein using the Protein Blast tool (https://blast.ncbi.nlm.nih.gov/Blast.cgi) among protein data bank (PDB) database (wwpdb.org). Finally, 4mn5 and 4mn6 PDB IDs were selected based on the coverage of the mutation site in VAN-S (residue 364) with 96.03 and 100% identifies, respectively, as wild-type templates for future protein analyses. 4mn5 and 4mn6 display the crystal structure of the PAS^CYTO^ domain from *S. aureus* WalK based on the X-ray diffraction method at 2 Å and 2.1 Å resolutions, respectively.

The DUET web server (http://biosig.unimelb.edu.au/duet/) was applied to predicting the effects of mutation H364R on the WalK protein stability.

DUET predicts the changes in protein stability upon the introduction of nsSNP using consolidating mCSM (mutation Cutoff Scanning Matrix) and SDM (Site Directed Mutator) computational approaches [37].

### Protein-Protein interactions analysis

Predicting the impact of mutation H364R on the protein-protein affinity of WalK was carried out by the mSCM-PPI2 web server (http://biosig.unimelb.edu.au/mcsm_ppi2/). The mSCM-PPI2 applied an optimized graph-based signature approach to better evaluate the molecular mechanism of the nsSNP using modelling the effects of variations in the inter-residue non-covalent interaction network [38].

### Protein-nucleic acid interactions analysis

Protein-DNA interaction changes upon mutation WalK-H364R were determined using mCSM-NAv2 web server (http://biosig.unimelb.edu.au/mcsm_na/), which predicts the effects of nsSNP in protein-coding regions on nucleic acid binding affinities [39].

### Prediction of the impact of nsSNP mutation on protein function

SIFT (sorts intolerant from tolerant) webserver (http://sift-dna.org) was used to predict changes in the protein function. SIFT performs PSI-BLAST search and predicts changes based on sequence homology and the physical properties of amino acid residues.

### Doubling time calculation

The doubling time was calculated using an obtained graph of the growth kinetic curve, as previously described with modifications [40]. Briefly, the *S. aureus* culture was adjusted to 0.01 at an optical density at 660 nm (OD_660_) in 20 ml BHIB and incubated for 15 h. The OD_660_ of these cultures was recorded every 1 h using a spectrophotometer (Biochrom WPA Biowave II, UK). The formula for calculating the doubling time (DT) was [(*t2* - *t1*) × log 2] / [log OD_660_ at *t2* - log OD_660_ at *t1*]. The abbreviations *t1* and *t2* are the start andend times of the exponential phase, respectively [40]. The experiment was performed in triplicates.

### Triton X-100 induced autolysis kinetic assay

20 ml of the *S. aureus* culture was collected by centrifugation (6800 × g for 10 min), washed twice with 20 ml of cold distilled water, and adjusted to an OD_600_ of 1 in 10 ml cold distilled water supplemented with 0.1% Triton X-100. The absorbance decline at 600 nm within 0, 1, 2, 3, 4, and 16 h of incubation time was measured (using a spectrophotometer) as autolysin activity [41]. The experiments were done on three independent occasions.

### Hemolysis assay

The α-hemolysin activity was examined as previously described [42], with minor modifications. The human RBCs were separated from plasma using centrifugation (900 × g for 2 min), washed three times with 1X PBS solution, and diluted (300 μl of RBCs in 10 ml PBS). 200 μl of *S.aureus* cells (OD_600_ = 0.3) added into diluted RBCs, incubated for 1 h (with shaking at 250 rpm), and centrifuged at 6000 × g for 10 min. In three independent experiments, the absorbance of the supernatant was measured (using the spectrophotometer) at OD_543_ nm and analyzed as hemolytic activity.

Triton X-100 and diluted RBCs in PBS without bacteria were used as positive and negative controls, respectively. The percent of hemolysis activity was calculated from the average of three experiments by the following formula: (A_543_ of the sample - A_543_ of negative control) / (A_543_ of positive control - A_543_ of negative control) × 100.

### Statistical analysis

SPSS24 statistical software (SPSS inc., Chicago, IL) was used for statistical analyses of data. A *P* value ≤0.05 was considered as a significant difference between the isogenic pair of VAN-S/VAN-I. The results were reported as mean ± standard deviation (SD).

The autolysis activity of the isogenic pair of VAN-S/VAN-I was compared using parametric paired-sample Student’s *t*-test. Data of hemolysis assay and doubling time were analyzed using the nonparametric two-tailed Wilcoxon signed-rank test.

The results of RT-qPCR were analyzed by ∆∆Ct method using the Relative Expression Software Tool (REST) 2009 (v2.0.13; Qiagen, Valencia, CA, USA).

SIFT predictions are based on the scores (Ranges: 0-1) and median sequence conservation (Ranges: 0-4.32; ideally: 2.75-3.5). The amino acid substitution was predicted deleterious if the score was ≤0.05 and tolerated if the score was ≥0.05. Median sequence conservation shows the diversity of the sequences used for prediction. If it was >3.25, there was a warning.

Predicted results of the DUET, mSCM-PPI2, and mCSM-NAv2 methods include the variation in Gibbs Free Energy (ΔΔG in kcal/mol).

## Supplementary Information

**Additional file 1.**

## Data Availability

The gene sequences from this study are available in the GenBank database (https://www.ncbi.nlm.nih.gov/) under the accession numbers of MN503664, MN503665, MN503668, and MN503669. Primers used in this study are described in the Supplementary data file online (Table S1).

## References

[1] Shariati A, Dadashi M, Moghadam MT, van Belkum A, Yaslianifard S, Darban-Sarokhalil D (2020). Global prevalence and distribution of vancomycin resistant, vancomycin intermediate and heterogeneously vancomycin intermediate Staphylococcus aureus clinical isolates: a systematic review and meta-analysis. Sci Rep.

[2] Baseri N, Najar-Peerayeh S, Amiri FB (2018). Prevalence of vancomycin-intermediate Staphylococcus aureus among clinical isolates in Iran: a systematic review and meta-analysis. J Glob Antimicrob Resist.

[3] Hu Q, Peng H, Rao X (2016). Molecular events for promotion of vancomycin resistance in vancomycin intermediate Staphylococcus aureus. Front Microbiol.

[4] Dubrac S, Boneca IG, Poupel O, Msadek T (2007). New insights into the WalK/WalR (YycG/YycF) essential signal transduction pathway reveal a major role in controlling cell wall metabolism and biofilm formation in Staphylococcus aureus. J Bacteriol.

[5] Dubrac S, Bisicchia P, Devine KM, Msadek T (2008). A matter of life and death: cell wall homeostasis and the WalKR (YycGF) essential signal transduction pathway. Mol Microbiol.

[6] Howden BP, McEvoy CR, Allen DL, Chua K, Gao W, Harrison PF, Bell J, Coombs G, Bennett-Wood V, Porter JL (2011). Evolution of multidrug resistance during Staphylococcus aureus infection involves mutation of the essential two component regulator WalKR. PLoS Pathog.

[7] Delauné A, Dubrac S, Blanchet C, Poupel O, Mäder U, Hiron A, Leduc A, Fitting C, Nicolas P, Cavaillon J-M (2012). The WalKR system controls major staphylococcal virulence genes and is involved in triggering the host inflammatory response. Infect Immun.

[8] Gardete S, Kim C, Hartmann BM, Mwangi M, Roux CM, Dunman PM, Chambers HF, Tomasz A (2012). Genetic pathway in acquisition and loss of vancomycin resistance in a methicillin resistant Staphylococcus aureus (MRSA) strain of clonal type USA300. PLoS Pathog.

[9] Monk IR, Shaikh N, Begg SL, Gajdiss M, Sharkey LK, Lee JY, Pidot SJ, Seemann T, Kuiper M, Winnen B (2019). Zinc-binding to the cytoplasmic PAS domain regulates the essential WalK histidine kinase of Staphylococcus aureus. Nat Commun.

[10] Fukushima T, Furihata I, Emmins R, Daniel RA, Hoch JA, Szurmant H (2011). A role for the essential YycG sensor histidine kinase in sensing cell division. Mol Microbiol.

[11] Werth BJ, Ashford NK, Penewit K, Waalkes A, Holmes EA, Ross DH, et al. Dalbavancin exposure in vitro selects for dalbavancin-non-susceptible and vancomycin-intermediate strains of methicillin-resistant Staphylococcusaureus. Clin Microbiol Infect. 2021;27(6):910-e1 10.1016/j.cmi.2020.08.025 PMC7914275 32866650

[12] Patel Y, Zhao H, Helmann JD (2020). A regulatory pathway that selectively up-regulates elongasome function in the absence of class A PBPs. Elife.

[13] Shaik NA, Bokhari HA, Masoodi TA, Shetty PJ, Ajabnoor GM, Elango R, et al. Molecular modelling and dynamics of CA2 missense mutations causative to carbonic anhydrase 2 deficiency syndrome. J Biomol Struct Dyn. 2020;38(14):4067–80. 10.1080/07391102.2019.1671899 31542996

[14] Munir A, Kumar N, Ramalingam SB, Tamilzhalagan S, Shanmugam SK, Palaniappan AN, Nair D, Priyadarshini P, Natarajan M, Tripathy S (2019). Identification and characterization of genetic determinants of isoniazid and rifampicin resistance in Mycobacterium tuberculosis in southern India. Sci Rep.

[15] Vedithi SC, Malhotra S, Das M, Daniel S, Kishore N, George A, Arumugam S, Rajan L, Ebenezer M, Ascher DB (2018). Structural implications of mutations conferring rifampin resistance in mycobacterium leprae. Sci Rep.

[16] Hawkey J, Ascher DB, Judd LM, Wick RR, Kostoulias X, Cleland H, et al. Evolution of carbapenem resistance in Acinetobacter baumannii during a prolonged infection. Microbial Genomics. 2018;4(3):e000165. 10.1099/mgen.0.000165 PMC5885017 29547094

[17] Pandurangan AP, Blundell TL (2020). Prediction of impacts of mutations on protein structure and interactions: SDM, a statistical approach, and mCSM, using machine learning. Protein Sci.

[18] Coates-Brown R, Moran JC, Pongchaikul P, Darby AC, Horsburgh MJ (2018). Comparative Genomics of Staphylococcus reveals determinants of speciation and diversification of antimicrobial defense. Front Microbiol.

[19] Shoji M, Cui L, Iizuka R, Komoto A, Neoh H-m, Watanabe Y, Hishinuma T, Hiramatsu K (2011). walK and clpP mutations confer reduced vancomycin susceptibility in Staphylococcus aureus. Antimicrob Agents Chemother.

[20] Prince A, Sandhu P, Ror P, Dash E, Sharma S, Arakha M, Jha S, Akhter Y, Saleem M (2016). Lipid-II independent antimicrobial mechanism of nisin depends on its crowding and degree of oligomerization. Sci Rep.

[21] Pinho MG, Errington J (2005). Recruitment of penicillin-binding protein PBP2 to the division site of Staphylococcus aureus is dependent on its transpeptidation substrates. Mol Microbiol.

[22] Zhu J, Liu B, Shu X, Sun B (2021). A novel mutation of walK confers vancomycin-intermediate resistance in methicillin-susceptible Staphylococcus aureus. Int J Med Microbiol.

[23] Traber KE, Lee E, Benson S, Corrigan R, Cantera M, Shopsin B, Novick RP (2008). agr function in clinical Staphylococcus aureus isolates. Microbiology (Reading, England).

[24] Hijikata A, Tsuji T, Shionyu M, Shirai T (2017). Decoding disease-causing mechanisms of missense mutations from supramolecular structures. Sci Rep.

[25] Cameron DR, Ward DV, Kostoulias X, Howden BP, Moellering RC, Eliopoulos GM, Peleg AY (2012). Serine/threonine phosphatase Stp1 contributes to reduced susceptibility to vancomycin and virulence in Staphylococcus aureus. J Infect Dis.

[26] van Hal SJ, Steen JA, Espedido BA, Grimmond SM, Cooper MA, Holden MT, Bentley SD, Gosbell IB, Jensen SO (2014). In vivo evolution of antimicrobial resistance in a series of Staphylococcus aureus patient isolates: the entire picture or a cautionary tale?. J Antimicrob Chemother.

[27] Ishii K, Tabuchi F, Matsuo M, Tatsuno K, Sato T, Okazaki M, Hamamoto H, Matsumoto Y, Kaito C, Aoyagi T (2015). Phenotypic and genomic comparisons of highly vancomycin-resistant Staphylococcus aureus strains developed from multiple clinical MRSA strains by in vitro mutagenesis. Sci Rep.

[28] Hafer C, Lin Y, Kornblum J, Lowy FD, Uhlemann A-C (2012). Contribution of selected gene mutations to resistance in clinical isolates of vancomycin-intermediate Staphylococcus aureus. Antimicrob Agents Chemother.

[29] McDougal LK, Steward CD, Killgore GE, Chaitram JM, McAllister SK, Tenover FC (2003). Pulsed-field gel electrophoresis typing of oxacillin-resistant Staphylococcus aureus isolates from the United States: establishing a national database. J Clin Microbiol.

[30] CLSI: M100 Performance Standards for Antimicrobial (2018). Clinical and Laboratory Standas Institute 950 West Valley Road, Suite 2500.

[31] Kim JW, Lee KJ. Single-nucleotide polymorphisms in a vancomycin-resistant Staphylococcus aureus strain based on whole-genome sequencing. Arch Microbiol. 2020;202(8):2255-61. 10.1007/s00203-020-01906-y PMC7455577 32535788

[32] Baseri N, Najar-Peerayeh S, Bakhshi B. The effect of subinhibitory concentration of chlorhexidine on the evolution of vancomycin-intermediate Staphylococcus aureus and the induction of mutations in walKR and vraTSR systems. Infect Genet Evol. 2021;87:104628. 10.1016/j.meegid.2020.104628 33171303

[33] Yin Y, Chen H, Li S, Gao H, Sun S, Li H, Wang R, Jin L, Liu Y, Wang H (2019). Daptomycin resistance in methicillin-resistant Staphylococcus aureus is conferred by IS256 insertion in the promoter of mprF along with mutations in mprF and walK. Int J Antimicrob Agents.

[34] Silveira A, Caierão J, Silva C, Anzai E, McCulloch J, d'Azevedo P, Sincero T (2019). Impact of mutations in hVISA isolates on decreased susceptibility to vancomycin, through population analyses profile–area under curve (PAP-AUC). Diagn Microbiol Infect Dis.

[35] Peng H, Hu Q, Shang W, Yuan J, Zhang X, Liu H, Zheng Y, Hu Z, Yang Y, Tan L (2017). WalK (S221P), a naturally occurring mutation, confers vancomycin resistance in VISA strain XN108. J Antimicrob Chemother.

[36] Berscheid A, François P, Strittmatter A, Gottschalk G, Schrenzel J, Sass P, Bierbaum G (2014). Generation of a vancomycin-intermediate Staphylococcus aureus (VISA) strain by two amino acid exchanges in VraS. J Antimicrob Chemother.

[37] Pires DE, Ascher DB, Blundell TL (2014). DUET: a server for predicting effects of mutations on protein stability using an integrated computational approach. Nucleic Acids Res.

[38] Rodrigues CH, Myung Y, Pires DE, Ascher DB (2019). mCSM-PPI2: predicting the effects of mutations on protein–protein interactions. Nucleic Acids Res.

[39] Pires DE, Ascher DB (2017). mCSM–NA: predicting the effects of mutations on protein–nucleic acids interactions. Nucleic Acids Res.

[40] Katayama Y, Sekine M, Hishinuma T, Aiba Y, Hiramatsu K (2016). Complete reconstitution of the vancomycin-intermediate Staphylococcus aureus phenotype of strain Mu50 in vancomycin-susceptible S. aureus. Antimicrob Agents Chemother.

[41] Beltrame CO, Cortes MF, Bonelli RR, de Almeida Correa AB, Botelho AMN, Americo MA, Fracalanzza SEL, Figueiredo AMS (2015). Inactivation of the autolysis-related genes lrgB and yycI in Staphylococcus aureus increases cell lysis-dependent eDNA release and enhances biofilm development in vitro and in vivo. PLoS One.

[42] Lee J-H, Kim Y-G, Ryu SY, Lee J (2016). Calcium-chelating alizarin and other anthraquinones inhibit biofilm formation and the hemolytic activity of Staphylococcus aureus. Sci Rep.

