# Supplementary material for: Investigating the effect of an identified mutation within a critical site of PAS domain of WalK protein in a vancomycin-intermediate resistant Staphylococcus aureus by computational approaches
Source: BMC Microbiol. 2021 Sep 2;21:240. doi: 10.1186/s12866-021-02298-9 (PMC8414773; doi:10.1186/s12866-021-02298-9)
Supplement: Supplementary file 1 — Additional file 1. [file 12866_2021_2298_MOESM1_ESM.docx]

Supplementary file

Investigating the effect of an identified mutation within a critical site of PAS domain of WalK protein in a vancomycin-intermediate resistant Staphylococcus aureus by computational approaches

Neda Baseri ^1^, Shahin Najar-Peerayeh ^1^, Bita Bakhshi ^1^*

^a^ Department of Bacteriology, Faculty of Medical Sciences, Tarbiat Modares University, Tehran, Iran

*Corressponding author: Bita Bakhshi ([b.bakhshi@modares.ac.ir](mailto:b.bakhshi@modares.ac.ir))

**Table S1 Primers used for qRT-PCR and sequencing experiments in this study**

| **Gene** | **Purpose** | **Primer sequence (5´→ 3´)** | **Reference** |
| --- | --- | --- | --- |
| ***walR*** | qRT-PCR | F: GGTATGGAAGTATGTCGTGAAGTG  R: TCTTGTGCTGGTTGTGAGTAATG |  |
| ***vraR*** | qRT-PCR | F: GAGTCGTCGCTTCTACAC  R: AAGTGATATTGAAGTAGTTGGTG | [[1](#_ENREF_1)] |
| ***graR*** | qRT-PCR | F: TGTTGCTGGTATTGAAGATTTCG  R: CATACTCATCACTTGATCCATTGG | [[1](#_ENREF_1)] |
| ***atlA*** | qRT-PCR | F: GCTGGTAGTGTGTCTGGCTCTG  R: TGATGGCTTAGGTGTTGGTGTAGG | [[1](#_ENREF_1)] |
| ***lytM*** | qRT-PCR | F: GATGGGCTTCGCTACATTTAC  R: ACATTGCTTGATTCCAGTTACC | [[1](#_ENREF_1)] |
| ***sle1*** | qRT-PCR | F: GGAATGCTAATAACTGGGATAAC  R: TGACCATAGTAACCTACATCTG | [[1](#_ENREF_1)] |
| ***sceD*** | qRT-PCR | F: GTGGTGCTTCAACTTCTT  R: CAACAGGTGCTAATGGAG | [[2](#_ENREF_2)] |
| ***RNA III*** | qRT-PCR | F: GAATTTGTTCACTGTGTCGATAATCCATTT  R: GAAGGAGTGATTTCAATGGCACAAGATAT | [[1](#_ENREF_1)] |
| ***gyrA*** | qRT-PCR | F: CGTGAAGGTGACGAAGTTGTAGG  R: TAACTGGCGTACGTTTACCATAACC | [[3](#_ENREF_3)] |
| ***walR*** | Sequencing | F: ATGGCTAGAAAAGTTGTTG  R: CTACTCATGTTGTTGGAGG | [[1](#_ENREF_1)] |
| ***walK*** | Sequencing | F1: ATGAAGTGGCTAAAACAACTAC  R1: GATGTCTTCTTTCGCCATAC  F2: GAAGACATCATCGGATATTACATG  R2: TTATTCATCCCAATCACCGTC | [[1](#_ENREF_1)] |

**
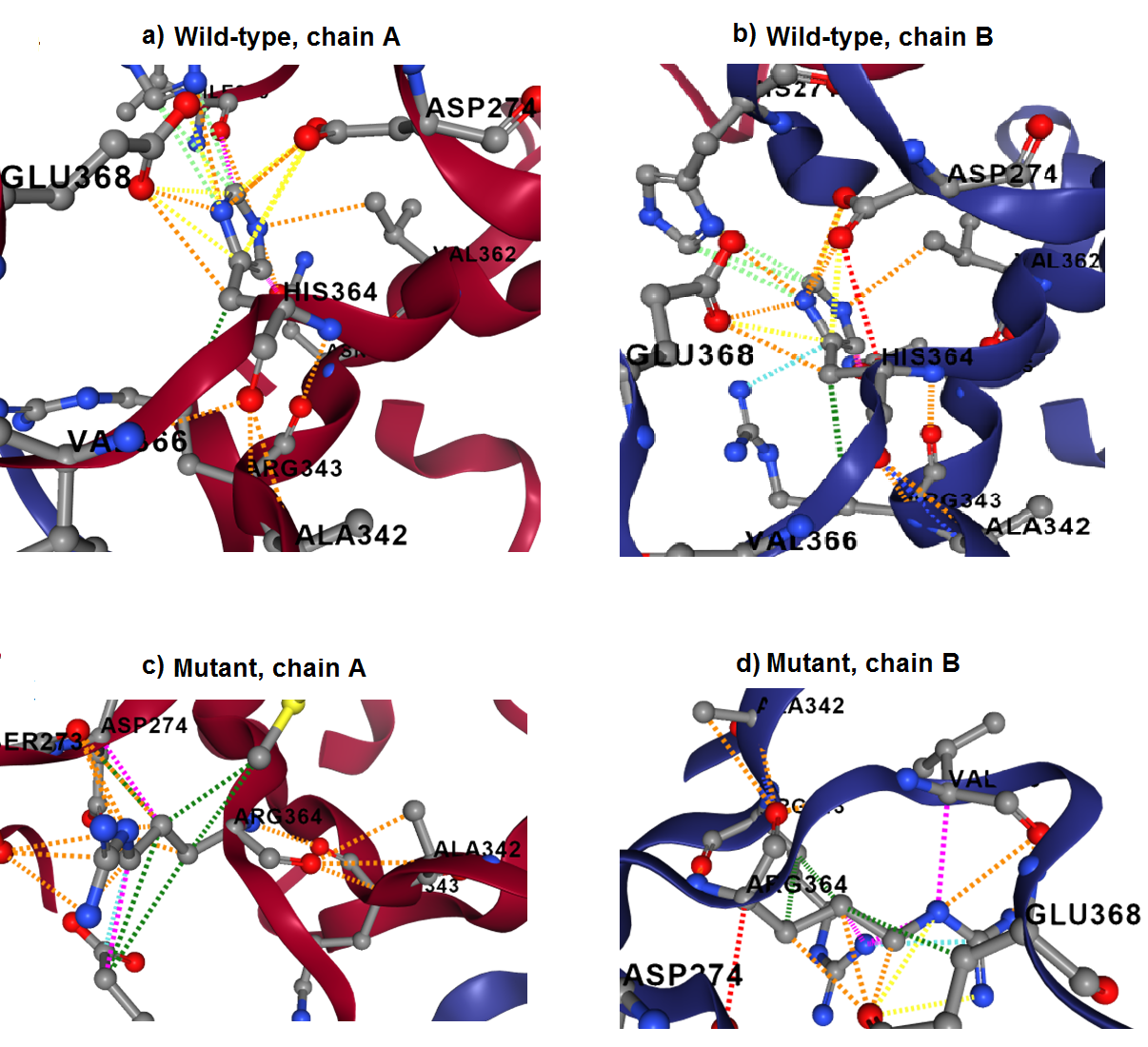
**

**Fig S1.** Three-dimensional (3D) view of the differences of interatomic interactions of HIS364 (wild-type) and ARG364 (mutant) residues in chain A and chain B of WalK in the mutant (VAN-I) and wildtype (PDB ID as a template: 4mn6). Binding interactions are reduced in the mutant than those in the wild-type. (a) Wild-type, chain A. (b) Wild-type, chain B. (c) Mutant, chain A. (d) Mutant, chain B. The color of interactions: clash: purple; VDM: pale blue; hydrogen bond: red; ionic: yellow; aromatic: pale green; hydrophobic: green; carbonyl: blue; polar: orange. The image is generated by the mCSM-PPI2 web server (<http://biosig.unimelb.edu.au/mcsm_ppi2/>).

**References:**

1. Baseri N, Najar-Peerayeh S, Bakhshi B: **The effect of subinhibitory concentration of chlorhexidine on the evolution of vancomycin-intermediate Staphylococcus aureus and the induction of mutations in walKR and vraTSR systems**. *Infection, Genetics and Evolution* 2020:104628.

2. Burian M, Rautenberg M, Kohler T, Fritz M, Krismer B, Unger C, Hoffman WH, Peschel A, Wolz C, Goerke C: **Temporal expression of adhesion factors and activity of global regulators during establishment of Staphylococcus aureus nasal colonization**. *The Journal of infectious diseases* 2010, **201**(9):1414-1421.

3. Rice KC, Firek BA, Nelson JB, Yang S-J, Patton TG, Bayles KW: **The Staphylococcus aureus cidAB operon: evaluation of its role in regulation of murein hydrolase activity and penicillin tolerance**. *Journal of bacteriology* 2003, **185**(8):2635-2643.
